# Vaginal Microbiome Composition, Diversity and Dysbiosis: The ORCHiD Study

**DOI:** 10.21203/rs.3.rs-7428423/v1

**Published:** 2025-09-11

**Authors:** April Deveaux, Oyomoare Osazuwa-Peters, Yeon Ji Kim, Pixu Shi, Drew Neish, Ashwini Joshi, Veronica Duck, Ashliegh Williams, Margaret Gates Kuliszewski, Bin Huang, Kevin Ward, Maria Pisu, Thomas Tucker, Rebecca Previs, Andrew Berchuck, Tomi Akinyemiju

**Affiliations:** Duke University School of Medicine; Duke University School of Medicine; Duke University; Duke University; Duke Cancer Institute; Duke University School of Medicine; Duke University School of Medicine; Duke Human Vaccine Institute; University at Albany; University of Kentucky; Emory University, Winship Cancer Institute; University of Alabama at Birmingham; University of Kentucky, Markey Cancer Center; Labcorp; Duke University; Duke University School of Medicine

**Keywords:** microbiome, ovarian cancer, ORCHiD, 16S rRNA sequencing, topic modeling, cancer disparities, Vaginal microbiome, topic modeling, health disparities, Lactobacillus

## Abstract

**Background:**

Vaginal dysbiosis may contribute to ovarian cancer (OC) outcomes, but comprehensive microbiome characterization remains limited. Here, we characterize the prevalence and predictors of vaginal microbiome dysbiosis among diverse OC patients in the US.

**Methods:**

We performed 16S rRNA gene sequencing on vaginal samples from 132 OC patients recruited as part of the population-based ORCHiD study. We applied topic modeling using Latent Dirichlet Allocation (LDA), a computational approach for identifying latent microbial community patterns, to identify distinct microbial signatures representing co-occurring bacterial taxa.

**Results:**

Widespread dysbiosis was observed with *Lactobacillus* detected in only 47.7% of patients. Topic modeling identified seven distinct microbial signatures from *Lactobacillus*-dominated to pathogenic communities. Patients ≥ 50 years showed significant anaerobic bacterial enrichment (log2FC = 1.31, FDR q < 0.001). Striking racial disparities emerged: Non Hispanic-Black patients had 5-fold higher Actinomycetaceae prevalence (40.9% vs 8.2%, FDR q = 0.005), while protective *L. crispatus* was detected exclusively in Non Hispanic-White patients (6.4% vs 0%).

**Conclusions:**

This study revealed widespread vaginal microbiome dysbiosis among OC patients with clinically significant age and racial patterns that may contribute to outcome disparities.

## Introduction

The vaginal microbiome, the community of microbes in the lower female genital tract, has emerged as an important factor in gynecologic health and gynecological cancers and may influence disease prognosis (1, 2). A healthy microbiome is typically dominated by *Lactobacillus* species, which help maintain a low pH and protect against pathogens(3, 4). When the protective *Lactobacillus*-dominated balance is disrupted—a state known as vaginal dysbiosis – the microbiome shifts toward communities dominated by anaerobic bacteria(4). This dysbiotic state has been linked to adverse vaginal health outcomes such as bacterial vaginosis, pelvic inflammatory disease and chronic genital inflammation(2, 5, 6), and can contribute to immune dysregulation, epithelial dysfunction, and pro-inflammatory signaling - important mechanisms in tumor progression(5, 7).

Ovarian cancer (OC) remains one of the most lethal gynecological malignancies in the United States, with a five-year survival rate of only 51%(8), however few studies have characterized the role of the vaginal microbiome in driving OC prognosis. Recent studies suggest that OC is associated with a shift in the vaginal microbiome toward a dysbiotic state(3, 5, 9). In a case-control study with ovarian cancer patients, Morikawa et al. found that the cervicovaginal microbiota of OC patients was characterized by significant loss of *Lactobacillus*-dominated communities and increased microbial diversity across multiple histologic subtypes compared to healthy controls(10). Sipos et al. proposed the concept of an “oncobiome”, describing how pathogen-driven disruptions such as infections with *Chlamydia trachomatis* or *Neisseria gonorrhoeae* may lead to lasting alterations in the vaginal microbiome that promote chronic inflammation and carcinogenesis(11). Building on this, Mehra et al. reviewed evidence of altered vaginal and gut microbial ecosystems in OC, reporting that *Lactobacillus* was present in only 24% of women with OC compared to 47% of healthy controls(12). They also identified dysbiosis-associated microbial shifts as contributors to immune dysregulation and tumor progression, suggesting the potential for microbiome modulation as a diagnostic or therapeutic strategy(12). Together, these findings describe a broader biological framework in which vaginal dysbiosis is both a marker and a possible mediator of OC pathophysiology.

The vaginal microbiome varies by patient demographics and clinical factors (13, 14). For instance, aging is associated with declining estrogen levels and shifts in immune function that reduce *Lactobacillus* abundance while increasing anaerobic bacterial diversity(15). The vaginal microbiome composition also varies by race/ethnicity (16), with documented differences in microbial diversity, bacterial species composition, and *Lactobacillus* distribution patterns(17–20). Such compositional differences might contribute to disparities in OC outcomes, including tumor aggressiveness and progression(5, 21, 22). Notably, Black women experience significantly worse OC outcomes compared to White women, including later-stage diagnosis, more aggressive tumor biology, and reduced survival(23, 24). While socioeconomic factors and healthcare access contribute to these disparities, emerging evidence suggests that biological factors, including microbiome differences, may also play a role(19, 25). A modest number of prior studies have investigated the role of the vaginal microbiome in OC outcomes (3, 26–28), however these investigations have largely evaluated alpha and beta diversity, differential abundance testing, and taxonomic profiling based on relative abundances(29, 30). These traditional analytical approaches, while informative, are unable to identify complex community-level patterns that emerge from co-occurring bacterial taxa(30, 31), insights that might reveal novel mechanistic pathways contributing to OC outcomes and disparities.

In this analysis, we aimed to characterize the vaginal microbiome composition, diversity and dysbiosis among patients in the Ovarian Cancer Epidemiology, Healthcare Access and Disparities Study (ORCHiD). Specifically, we sought to: (1) describe the study population characteristics and overall microbiome diversity patterns in OC patients; (2) characterize vaginal microbiome composition using traditional differential abundance analysis across patient demographics and disease characteristics; (3) identify distinct vaginal microbial community signatures using topic modeling and examine their associations with clinical variables; and (4) validate findings through targeted species-level analysis of key *Lactobacillus* species. Our findings may contribute to understanding microbiome-mediated health disparities and inform targeted therapeutic interventions aimed at improving OC outcomes across demographic groups.

## Methods

### Study Design and Population

**Figure 1:**
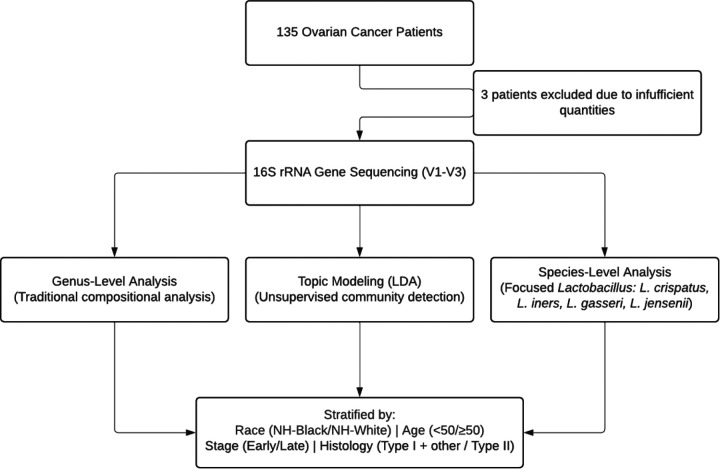
Study Design and Multi-Scale Analytical Framework for Dysbiotic Populations. Flow chart illustrating the analytical approach used in the ORCHiD study. A total of 132 OC patients provided vaginal samples for 16S rRNA gene sequencing (V1-V3 region). Three complementary analytical approaches were employed: traditional genus-level analysis, topic modeling using Latent Dirichlet Allocation (LDA) for unsupervised community detection, and species-level analysis focused on *L. crispatus, L. iners, L. gasseri,* and *L. jensenii*. All analyses were stratified by self-reported race (NH-Black/NH-White), age (<50/≥50 years), cancer stage (Early/Late), and histology Type I + Other/Type II).

An overview of the study design and framework is presented in Fig. 1. This study included 135 ovarian cancer (OC) patients recruited as part of the ORCHiD study. Detailed methods are published elsewhere(32). Briefly, this population-based study recruited patients with confirmed first primary OC between March 2021 and October 2024 from seven state cancer registries, including New York, Kentucky, California, North Carolina, Georgia, and Texas. The study inclusion criteria were patients aged 18 years and older with a pathologically confirmed diagnosis of OC (stages I-IV) at 9–12 months prior to recruitment. All patients in this cohort self-identified their race/ethnicity as non-Hispanic Black (NH-Black) or non-Hispanic White (NH-White). The study exclusion criteria were patients older than 79 years old, or patients with cognitive impairments preventing survey completion. The ORCHiD survey comprehensively captures data on patient demographics, socio-economic status, healthcare access, as well as self-reported treatment, lifestyle, and health history data. Eligible patients were contacted via phone to confirm eligibility, conduct informed consent, and determine participation interest. Patients who consent to participate complete the ORCHiD survey over the phone, online, or by mail and receive an incentive of $25 for survey completion.

### Vaginal Microbiome Sample Collection and Processing

Patients who completed the ORCHiD survey were offered an opportunity to participate in the ORCHiD biospecimen sub study which required self-collection of both a vaginal swab and saliva. Once a patient consented to the sub-study, the study team mailed a biospecimen packet containing a vaginal microbiome collection kit (OMR-130, DNA Genotek, Ottawa, Canada) along with manufacturer instructions and a postage-paid sample return envelope. Each biospecimen packet also included a brief 4-question behavioral survey querying medications or microbiome-altering behaviors in the previous 30 days, including oral or IV antibiotic use or use of douching or suppository products. Patients responding yes to any of the microbiome survey questions were excluded from the analysis cohort. Patients were also instructed not to collect samples when actively menstruating or experiencing other vaginal bleeding. Upon receipt of returned kits, samples were inspected for any damage or leakage and logged electronically. The collection kit included a swab and a vial containing microbial media buffer, a proprietary stabilizing liquid to preserve samples at room temperature for up to 30 days. Any damaged kits were disposed of according to established safety regulations. Samples were processed by treating with 5 μl of Proteinase K (80 mg/ml) and incubating for one hour in a 50°C water bath, then aliquoted into 1.5 ml microcentrifuge tubes and stored at −80°C for downstream processing. Of the 764 patients recruited into the parent ORCHiD study, 435 (56.9%) consented to the biospecimen sub-study, and 230 (52.9% of consenters, 30.1% of total surveyed) returned at least one sample. Patients who completed and returned the biospecimen kits received an additional $20 incentive.

Processed vaginal samples were sent to the Duke Microbiome Core Facility for assay. First, microbial DNA from vaginal swab samples preserved in OMNIgene vaginal kit was extracted using a DNeasy 96 PowerSoil Pro QIACube HT kit (QIAGEN, #47021) on an automated machine (QIACube HT, QIAGEN). The manufacturer’s instructions were followed with these minor deviations: 250 μl of the preserved sample was used as starting material, and during the final elution step, the elution buffer (C6) was incubated at room temperature for 10 minutes to increase DNA yield. Bacterial community composition from vaginal swab samples was characterized by amplification of the 16S rRNA V1-V3 hypervariable regions via polymerase chain reaction (PCR). Forward primer 27F (5’-GAGTTTGATCGTGGCTCAG-3’) and reverse primer 518R (5’-ATTACCGCGGCTGCTGG-3’) were used with Phusion Plus PCR Master Mix (ThermoScientific, #F631L) for a total of 25 PCR cycles. Samples were multiplexed via the reverse primers (518R) that carry unique Earth Microbiome Project (http://www.earthmicrobiome.org/) barcodes (33). PCR products were purified with AMPure XP Beads (Beckman Coulter, #A63881) and quantified with a Qubit dsDNA HS assay kit (ThermoFisher, #Q32854) on a Promega GloMax plate reader. Equimolar PCR products were pooled from each sample and submitted to the Duke Sequencing and Genomic Technologies shared resource. The final pool was sequenced on an Illumina MiSeq platform using v3 chemistry (300 base pairs, paired-end).

### Study Variables

Cohort characteristics assessed included self-reported race, age at diagnosis, OC stage, and histologic subtype. Race was self-reported and categorized as non-Hispanic Black or non-Hispanic White. Age was analyzed as a continuous variable for primary analyses and then dichotomized into two age categories (< 50 years and ≥50 years) for interpretability of topic distributions. Clinical data, such as stage at diagnosis and histological subtype, were obtained from state cancer registries. OC stage was derived from summary staging data and categorized into early-stage (summary stage 1 or 2), or late-stage (summary stage greater than 2). Histologic subtype was categorized as either type II epithelial (including high-grade serous and other aggressive subtypes) or Other (including type I epithelial, endometrioid, mucinous, clear cell subtypes, and non-epithelial tumors).

### Statistical Analysis

#### Data Processing

Microbiome sequencing data was processed using R programming suite (v4.3.0) (34). Sequencing reads from vaginal samples were processed using the DADA2 pipeline (v1.28.0) (35) to infer amplicon sequence variants (ASVs) with default parameters. Deviations from default parameters include a parameter for FilterAndTrim function (truncLen = c(290, 260)) to remove sequencing regions with low quality scores and only including ASVs that were 433 to 525 base pairs long in analysis. Taxonomy was assigned using the Silva v138.1 database (36, 37) and non-bacterial taxa were removed. Samples with less than 1,000 reads were removed, which included 4 negative controls and 3 samples with insufficient sample size, and a phylogenetic tree was constructed using Randomized Axelerated Maximum Likelihood (RAxML) to account for phylogenetic relationships (38).

##### Summary Statistics:

For taxonomic composition analysis, we calculated three key metrics for each bacterial genus: (1) prevalence, defined as the percentage of samples in which the genus was detected above the minimum threshold; (2) mean relative abundance, calculated as the average percentage of total reads attributed to each genus across all samples; and (3) median relative abundance with interquartile range (IQR), providing robust measures of central tendency and spread for non-normally distributed abundance data.

##### Topic Modeling Analysis:

We employed Latent Dirichlet Allocation (LDA) to identify distinct vaginal microbial community signatures, termed “microbial topics,” representing co-occurring bacterial taxa(39). The range of 3 to 10 topics was selected based on established vaginal microbiome community classifications, as the foundational community state type (CST) framework identifies 5 CSTs with up to 9 CSTs with extended classification(16, 40). This biologically informed range ensures alignment with known vaginal microbiome structure while allowing for potential novel community patterns in this cancer population. Optimal topic number was determined using the FindTopicsNumber function from the “ldatuning” R package (v1.0.2)(41) with multiple metrics including CaoJuan2009, Arun2010, Griffiths2004, and Deveaud2014 (42–45) evaluated across the topic number range. Two metrics (CaoJuan2009 and Arun2010) identify optimal models by minimization, while the other two metrics (Griffiths2004 and Deveaud2014) identify optimal models by maximization. The optimal number of topics was selected using an elbow method approach (**Supplemental Fig. 1**). LDA was implemented using the “topicmodels” package (v0.2.17) in R(46) with Gibbs sampling method, treating samples as “documents” and genera as “words.” The model was run with a fixed seed (243) for reproducibility. Beta probability matrices (genus-topic associations) and gamma probability matrices (sample-topic associations) were extracted using the “tidytext” package (v0.4.3) (47). Topics were characterized by their dominant genera (highest beta probabilities) and named based on their microbial composition and established associations with vaginal health states.

##### Differential Abundance Analysis of Topics:

Topic-level differential abundance testing was performed using the LinDA (linear models for *differential* abundance analysis) framework(48), which accounts for compositional data structure and provides robust statistical inference. Topic counts were calculated by multiplying gamma probabilities by corresponding read counts for each sample, creating a topic-level phyloseq object. LinDA analysis was conducted with the formula: ~ Race + Stage + Histology + Age, testing each clinical variable while controlling for others. The analysis used empirical Bayes shrinkage, centered log-ratio transformation, and Benjamini-Hochberg false discovery rate (FDR) correction for multiple testing (FDR q < 0.05).

##### Species-Level Lactobacillus Validation:

To validate topic-level findings, we performed targeted specieslevel analyses focusing on key *Lactobacillus* species known to be important for vaginal health. The phyloseq object was filtered to include only *Lactobacillus* species, and relative abundances were calculated by dividing species counts by total reads per sample. We examined both detection rates (presence/absence) and relative abundances when present for *L. crispatus*, *L. iners*, *L. gasseri*, and *L. jensenii* across demographic and clinical variables.

##### Additional Analyses:

Detection rates between groups were compared using Fisher’s exact tests, while relative abundances (when detected) were compared using Wilcoxon rank-sum tests due to non-normal distributions. For detection rate analyses, effect sizes were calculated as odds ratios with 95% confidence intervals. For abundance comparisons, effect sizes were reported as fold-changes between group medians. FDR correction was applied across all species tested within each comparison type (detection vs abundance). Statistical significance was set at FDR q < 0.05, with trends reported for FDR q < 0.10. Alpha diversity (Shannon diversity index and Faith’s phylogenetic distance) was calculated using phyloseq (v1.44.0) and picante (v1.8.2) packages, while beta diversity was assessed using Jaccard, Bray-Curtis, UniFrac, and weighted UniFrac dissimilarity metrics calculated with the vegan package (v2.6.4), with principal coordinate analysis (PCoA) performed for visualization. Alpha diversity metrics were tested for normality using Shapiro-Wilk tests, and differences by demographic and clinical variables were assessed using appropriate parametric (ANOVA) or non-parametric (Kruskal-Wallis) tests, while beta diversity differences were tested using permutational analysis of variance (PERMANOVA) with FDR correction applied across multiple comparisons.

##### Data Visualization and Statistical Software:

All statistical analyses were performed in R (v4.3.0)(49) using the following packages: *phyloseq* (v1.48.0) for microbiome data handling(50), *topicmodels* (v0.2.17) for LDA implementation(46), *tidytext* (v0.4.3) for topic model interpretation(47), *LinDA* (v0.2.0) for differential abundance testing(48), *ggplot2* (v3.5.2)for visualization(51), and *cowplot* (v1.2.0)for multi-panel figures(52). Statistical significance was set at FDR-adjusted q < 0.05 unless otherwise specified.

## Results

### Cohort Characteristics

A total of 132 OC patients were included in the analysis (Table 1); 83.7% NH-White (n = 111) and 16.3% NH-Black (n = 22). The median age was 61.5 years (range 30–79), with 83.0% of patients 50 years or older at diagnosis. Compared to NH-White patients, NH-Black patients were more likely to report lower annual household income (22.7% vs. 7.1% reporting <$20,000), unemployment (13.6% vs. 3.5%), or disability (27.3% vs. 2.7%). About 72.7% of NH-Black patients widowed, divorced, separated, or never married, compared to 31.9% of NH-White patients. Most patients were diagnosed with late-stage disease, defined as FIGO stage III–IV (67.4%), although late-stage diagnosis was higher in NH-Black vs NH-White patients (77.3% vs. 65.5%). Type II epithelial was the most common histologic subtype (60.0%). Most patients received surgery (94.1%) and chemotherapy (75.6%), with no notable differences in treatment by race.

### Microbiome Diversity

We evaluated vaginal microbiome diversity using both alpha (Shannon index) and beta (weighted UniFrac distance) diversity metrics (**Supplemental Fig. 1**). Alpha diversity, assessed via the Shannon index which incorporates both richness and evenness, did not differ significantly by race (median: 2.37 NH-Black vs. 2.52 NH-White; p = 0.72; **Figure S1A**), OC stage (median: 2.54 early vs. 2.42 late; p = 0.49; **Figure S1C**), or histologic subtype (median: 2.39 Type I vs. 2.48 Type II; p = 0.78; **Figure S1E**). These findings suggest that within-sample microbial richness and evenness were relatively stable across demographic and clinical strata. Beta diversity, quantified using weighted UniFrac distances which incorporates relative abundance and phylogenetic information, showed no significant differences in overall microbial community composition by race (PERMANOVA p = 0.64; **Figure S1B**) or stage (PERMANOVA p = 0.76; **Figure S1D**). However, significant differences were observed by histologic subtype (PERMANOVA p = 0.025; **Figure S1F**), indicating that the structure of the vaginal microbiome varied between Type I and Type II epithelial OC.

### Vaginal Microbiome Composition and Relative Abundance

**Figure 2:**
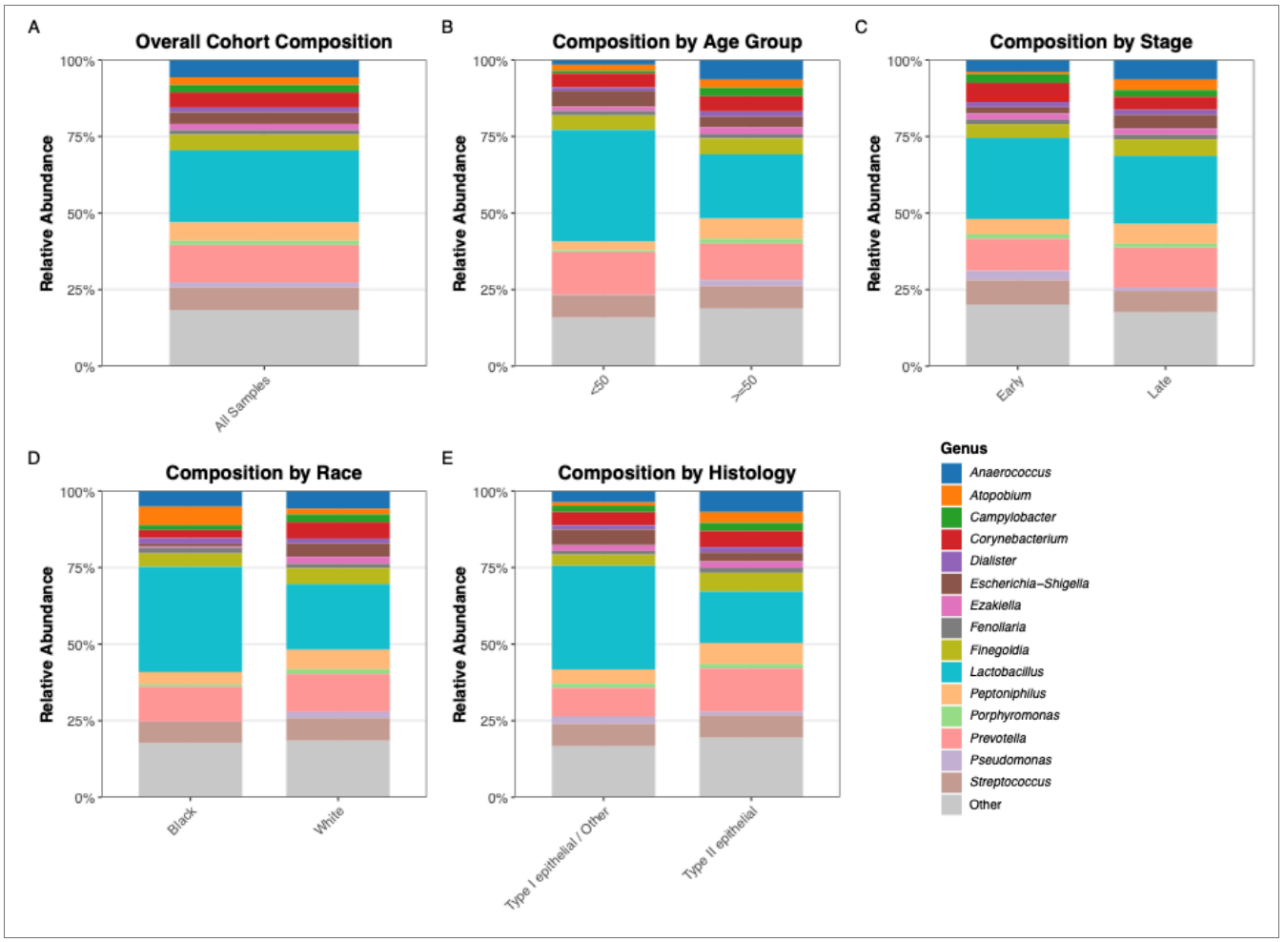
Vaginal Microbiome Composition Across Demographic and Clinical Variables. Stacked bar charts showing relative abundance of major bacterial genera in OC patients. (A) Overall cohort composition, (B) Composition by age group (<50 vs ≥50 years, (C) Composition by cancer stage (Early vs Late), (D) Composition by race (NH-Black vs NH-White), and (E) Composition by histologic subtype (Type I epithelial + Other vs Type II epithelial).

Vaginal microbiome overall composition analysis (Fig. 2, **Supplemental File**) showed *Lactobacillus* as the most common genus by mean relative abundance (mean: 23.5%, median: 0% [IQR: 0–44.5%], prevalence: 47.7%), followed by *Prevotella* (mean: 12.3%, median: 4.9% [IQR: 0.6–23.6%], prevalence: 93.9%) and other anaerobic genera including *Peptoniphilus* (mean: 6.0%, prevalence: 95.5%), *Anaerococcus* (mean: 5.6%, prevalence: 90.9%), and *Finegoldia* (mean: 5.1%, prevalence: 96.2%). The difference between mean and median abundance for *Lactobacillus* (23.5% vs. 0%) indicated a bimodal distribution, with this genus being either absent/minimal or highly dominant across individuals. This pattern reflects a generally dysbiotic microbiome across the cohort, with *Lactobacillus* species present in less than half of patients and anaerobic bacteria widely prevalent (> 90% prevalence for most genera).

### Age-Related Differences

Older patients ≥ 50 years had significantly higher prevalence of *Actinotignum* compared to younger patients (< 50 years: prevalence 31.8%; ≥50 years: prevalence 68.2%, FDR q = 0.007), though mean relative abundances remained low in both groups (< 50: mean 2.0%, median 0%; ≥50: mean 2.7%, median 0%). This age-related pattern suggests increased colonization by this genus in older OC patients. There were no other significant or trending associations by age group.

### Cancer Stage Differences

Early stage patients had significantly higher prevalence of *Cutibacterium* compared to late-stage patients (prevalence: 61.0% vs 33.0%, FDR q = 0.048). There were no other significant or trending associations by disease stage.

### Racial Differences

NH-Black patients had a 5-fold higher prevalence of Actinomycetaceae family bacteria compared to NHWhite patients (40.9% vs 8.2%, FDR q = 0.005), representing the most robust compositional difference observed in the cohort. NH-Black patients also had higher *Lactobacillus* prevalence (68.2% vs 43.6%) and mean abundance (mean 34.4% vs 21.4%), though this did not reach statistical significance (FDR q = 0.271). Notably, while overall *Lactobacillus* abundance appeared higher in NH-Black patients, this pattern was driven primarily by different species composition (detailed in species-level analysis below). There were no other significant or trending racial differences in microbial composition.

### Histologic Subtype Differences

There were no significant or trending associations between microbial composition and histologic subtype (all FDR q > 0.25).

### Topic Modeling Results Identification of Vaginal Microbial Topics

**Figure 3:**
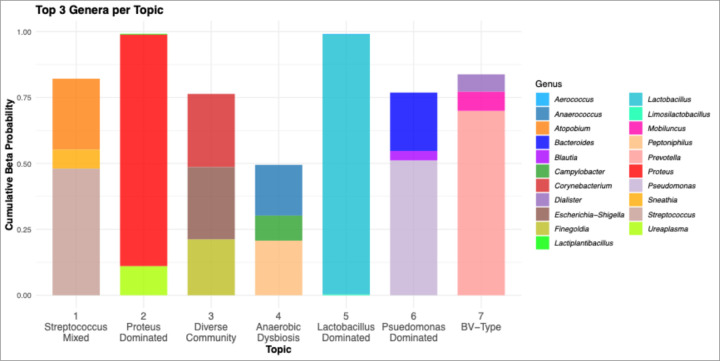
Vaginal Microbiome Topic Signatures Identified by Latent Dirichlet Allocation. **Bar chart displaying** the top 3 genera per microbial topic based on beta probability values. Seven distinct topics were identified: Topic 1 (*Streptococcus*Mixed) - moderate dysbiosis characterized by *Streptococcus* (48.1%), *Atopobium*(26.9%), and *Sneathia* (7.2%); Topic 2 (*Proteus* Dominated) - severe pathogenic dysbiosis dominated by *Proteus* (87.9%); Topic 3 (Diverse Community) - intermediate dysbiosis with *Corynebacterium*(27.8%), *Escherichia-Shigella* (27.5%), and *Finegoldia* (21.2%); Topic 4 (Anaerobic Dysbiosis) - anaerobic dysbiosis led by *Peptoniphilus*(20.7%) and *Anaerococcus* (19.3%); Topic 5 (*Lactobacillus*Dominated) - healthy signature nearly exclusively composed of *Lactobacillus*(98.6%); Topic 6 (*Pseudomonas* Dominated) - potentially representing opportunistic growth with *Pseudomonas* (51.2%); Topic 7 (BV-Type) - bacterial vaginosis-type dysbiosis dominated by *Prevotella* (70.0%).

LDA identified seven microbial topics using an elbow method approach, representing distinct cooccurring genera patterns that were biologically interpretable without excessive model complexity(53) (Fig. 3, **Supplemental Fig. 2).** Topic 1 (*Streptococcus* Mixed) was characterized by moderate dysbiosis with *Streptococcus* (48.1%), *Atopobium* (26.9%), and *Sneathia* (7.2%), as *Atopobium* and *Sneathia* are established bacterial vaginosis-associated taxa(16, 19). Topic 2 (*Proteus* Dominated) represented severe pathogenic dysbiosis, being highly dominated by *Proteus* (87.9%) with minimal diversity, as *Proteus* species are associated with urogenital infections and pathogenic overgrowth(54). Topic 3 (Diverse Community) showed intermediate dysbiosis led by *Corynebacterium* (27.8%), *Escherichia-Shigella* (27.5%), and *Finegoldia* (21.2%), representing the diverse, non-*Lactobacillus* communities characteristic of dysbiotic states(16). Topic 4 (Anaerobic Dysbiosis) was dominated by *Peptoniphilus* (20.7%) and *Anaerococcus* (19.3%) with *Campylobacter* (9.5%), representing anaerobic dysbiosis as these taxa are associated with bacterial vaginosis and inflammatory conditions(55). Topic 5 (*Lactobacillus*-Dominated) was nearly exclusively composed of *Lactobacillus* (98.6%), which can vary significantly in protective capacity depending on the specific species composition(16). Topic 6 (*Pseudomonas*-dominated) was led by *Pseudomonas* (51.2%) with diverse anaerobes that can represent opportunistic bacterial overgrowth in severely dysbiotic vaginal environments(56). Topic 7 (BV-Type) was dominated by *Prevotella* (70.0%), representing classic bacterial vaginosis-type dysbiosis as *Prevotella* is a hallmark of BV-associated microbiomes(16, 19).

### Topic-Clinical Variable Associations

**Figure 4:**
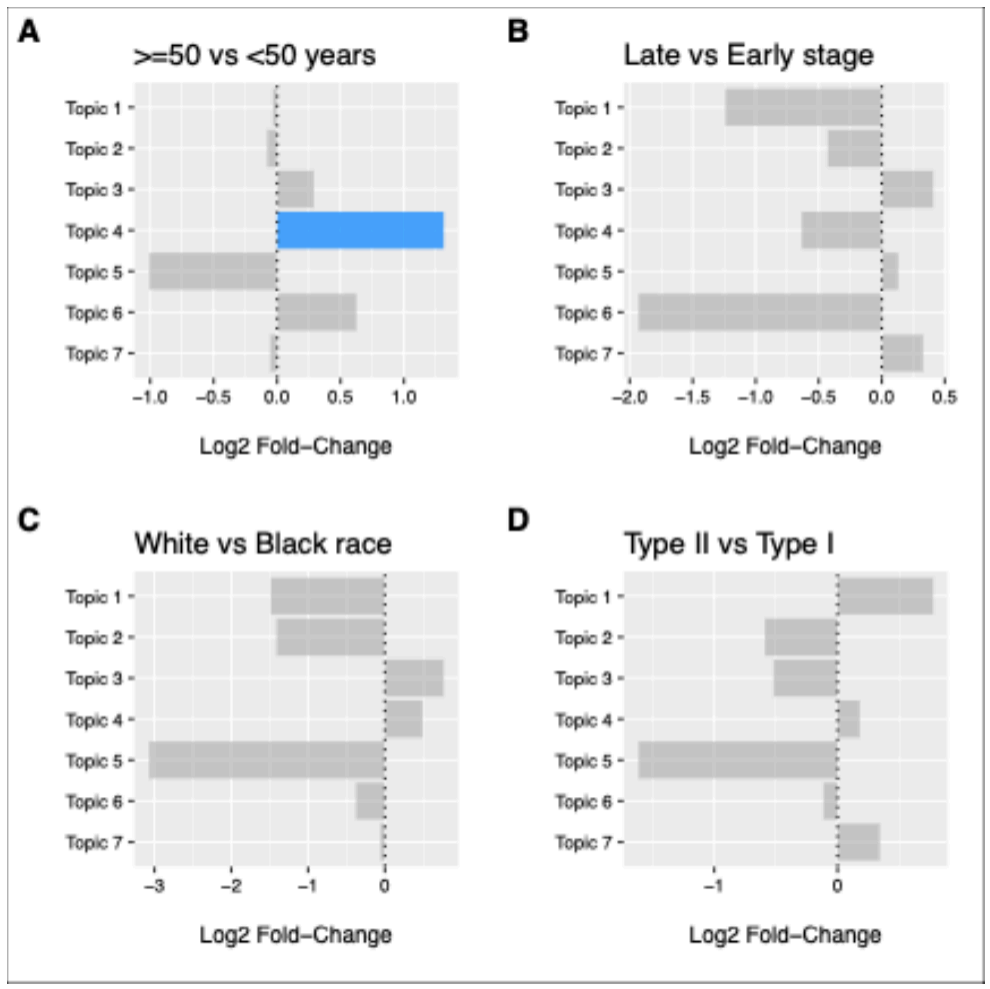
Topic-Clinical Variable Associations Using LinDA Differential Abundance Analysis. Forest plots showing log2fold-changes for microbial topics across clinical variables. (A) Age comparison (≥50 vs <50 years) revealing Topic 4 (Transitional/Anaerobic Dysbiosis) as significantly enriched in older patients (log_2_ FC = 1.31, FDR p = 0.001, highlighted in blue). (B) Disease stage comparison (Late vs Early) demonstrating Topic 7 (BV-Type) enrichment in late-stage disease (log2 FC = −1.5). (C) Race comparison (White vs Black) showing Topic 5 (*Lactobacillus*-dominated) depletion in White patients (log2FC = −2.5). (D) Histologic subtype comparison (Type II vs Type I) showing variable topic distributions across cancer types. Gray bars indicate non-significant associations, while the blue bar represents the only statistically significant finding after FDR correction.

LinDA differential abundance analysis revealed significant associations between microbial topics and patient demographics (Fig. 4). Topic 4 (Anaerobic Dysbiosis) showed the strongest and only statistically significant association, with significantly higher abundance in patients ≥ 50 years compared to younger patients (log2 fold-change = 1.31, FDR q < 0.001). This was the only topic-clinical variable association reaching statistical significance after multiple testing correction. Disease stage showed limited associations with topic abundance after multiple testing correction. Topic 6 (Environmental) showed enrichment in late-stage compared to early-stage disease (log2 fold-change = −1.9, FDR q = 0.06), though this did not reach statistical significance. While no racial associations reached statistical significance after FDR correction, consistent directional patterns emerged across topics. Topic 5 (*Lactobacillus*-dominated) showed the most substantial depletion in NH-White compared to NH-Black patients (log_2_ fold-change = −3.0, FDR q = 0.09), and Topic 2 (*Proteus*-Dominated) demonstrated depletion in NH-White versus NH-Black patients (log2 fold-change = −1.4, FDR q = 0.09). No significant associations were observed between topics and histologic subtypes after FDR correction, suggesting that overall microbial community patterns may be more influenced by demographic factors than tumor characteristics.

### Species-Level Analysis of Key Lactobacillus Species

**Figure 5:**
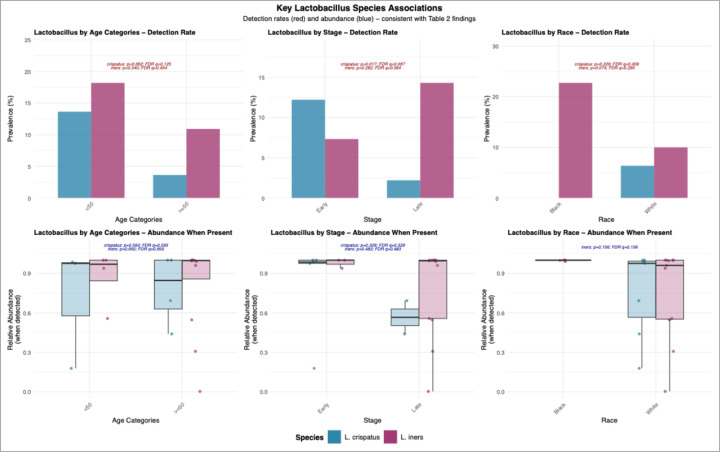
Species-Level Lactobacillus Validation of Topic Modeling Findings. Analysis of key *Lactobacillus* species supporting topic-level results. Top panels show detection rates (prevalence) for *L. crispatus* (teal) and *L. iners* (purple) by age category, cancer stage, and race. *L. crispatus* detection was 3.75-fold higher in younger vs older patients (13.6% vs 3.6%, FDR p = 0.125) and 5.55-fold higher in early-stage vs late-stage patients (12.2% vs 2.2%, FDR p = 0.067), while being detected exclusively in White patients (6.4% vs 0% in Black patients, FDR p = 0.458). Conversely, *L. iners* showed 2.27-fold higher prevalence in Black vs White patients (22.7% vs 10%, FDR p = 0.295). Bottom panels display relative abundance when species were detected, showing that *L. crispatus* maintains high abundance levels when present, while *L. iners* shows more variable patterns.

To validate the topic modeling findings, particularly regarding the healthy *Lactobacillus* signature (Topic 5), we performed detailed species-level analyses of the four predominant vaginal *Lactobacillus* species (**Supplemental File**) with key findings for *L. crispatus* and *L. iners* highlighted in Fig. 5. Given the bimodal distribution of *Lactobacillus* observed at the genus level, we examined both detection rates (presence/absence) and relative abundance when present for key *Lactobacillus* species.

### Detection Rates and Clinical Associations

Age-related patterns revealed differential effects on *Lactobacillus* species colonization. *L. crispatus* detection was 3.75-fold higher in patients < 50 years compared to those ≥ 50 years (13.6% vs 3.6%, FDR p = 0.125), while *L. iners* showed a similar but less pronounced age-related decline (18.2% vs 10.9%, FDR p = 0.454). *L. crispatus* was detected in 12.2% of early-stage patients compared to only 2.2% of late-stage patients, representing a 5.55-fold difference (FDR p = 0.067). In contrast, *L. iners* showed the opposite pattern, with higher detection in late-stage patients (14.3%) compared to early-stage patients (7.3%, FDR p = 0.389). Racial differences revealed striking opposing patterns in Lactobacillus species distribution. *L. crispatus* was detected exclusively in NH-White patients (6.4% vs 0% in NH-Black patients, FDR p = 0.458), while *L. iners* showed 2.27-fold higher prevalence in NH-Black patients (22.7% vs 10.0%, FDR p = 0.295).

### Abundance When Present

Among samples where *Lactobacillus* species were detected, abundance levels showed distinct patterns across clinical groups. When present, *L. crispatus* maintained consistently high relative abundance regardless of clinical characteristics. In early-stage patients, *L. crispatus* achieved a median relative abundance of approximately 95% when detected, with significantly higher abundance compared to late-stage patients (FDR p = 0.033). Age-related differences in *L. crispatus* abundance were minimal when the species was present (FDR p = 0.125). In contrast, *L. iners* demonstrated more variable abundance patterns when present. Abundance levels showed less consistent patterns across clinical groups, with median values ranging from 88–92% depending on disease stage and patient characteristics (all FDR p > 0.05). This variability may reflect the intermediate protective capacity of *L. iners* compared to the more robustly protective *L. crispatus*.

## Discussion

This comprehensive analysis of 132 OC patients identified consistent vaginal microbiome dysbiosis characterized by *Lactobacillus* depletion and anaerobic bacterial overgrowth. We observed two clinically significant findings: age-related increases in anaerobic dysbiosis, with patients ≥ 50 years showing significant enrichment of *Peptoniphilus* and *Anaerococcus* (log2 fold-change = 1.31, FDR q < 0.001), and striking racial disparities including a 5-fold higher prevalence of Actinomycetaceae family bacteria in NH-Black compared to NH-White patients (40.9% vs 8.2%, FDR q = 0.005). Species-specific *Lactobacillus* analyses revealed *L. crispatus* detected exclusively in NH-White patients and *L. iners* over 2-fold higher in NH-Black patients, alongside progressive loss of protective *L. crispatus* with advancing age and disease stage. These patterns suggest that the vaginal microbiome may play an important role in driving OC prognosis and health disparities, warranting further investigation for microbiome-based interventions.

These findings align with and extend established literature on vaginal microbiome alterations in gynecological malignancies. Previous OC studies by Jacobson et al. (2021) and Asangba et al. (2023) reported *Lactobacillus* depletion and anaerobic overgrowth, with only 24% of OC patients showing *Lactobacillus*-dominated microbiomes and significantly reduced abundance compared to controls (approximately 15% versus 30%)(27, 57). Our cohort showed similar patterns with 47.7% *Lactobacillus* detection and 23.5% mean relative abundance, falling substantially below healthy reproductive-age women rates (70–80% dominance)(16). However, our analysis also revealed age-specific patterns of dysbiosis, highlighting that the vaginal microbiome of older OC patients was significantly enriched with *Peptoniphilus* and *Anaerococcus* (log2 fold-change = 1.31, FDR p < 0.001), a notable finding that has not been previously reported to our knowledge. The racial differences we observed also extends prior work by Ravel et al., which established that healthy Black women are more likely to have non-*Lactobacillus*dominated communities, with only 61.9% exhibiting *Lactobacillus*-dominated communities compared to 89.7% of White women(16). However, no known studies have specifically examined racial differences in vaginal microbiome composition among OC patients. We observed that Topic 5 (*Lactobacillus*-dominated) showed substantial depletion in NH-White compared to NH-Black OC patients (log_2_ fold-change = −3.0, FDR q = 0.09), suggesting higher overall *Lactobacillus* prevalence in NH-Black OC patients that is worth further exploration as a potential OC-related signature. However, species-level validation revealed that this pattern is driven mainly by the predominance of dysbiotic *L. iners*, which was over 2-fold higher in NH-Black vs. NH-White patients (22.7% vs 10.0%), rather than the protective *L. crispatus* that was detected exclusively in White patients (6.4% vs 0%). Additionally, our study is the first to document that NH-Black OC patients have a 5-fold higher Actinomycetaceae family prevalence vs. White patients, findings that can be further explored in larger cohort sizes.

The age-related enrichment of *Peptoniphilus*-dominated signatures in our study reveals potentially important biological pathways linking aging, microbiome dysbiosis, and cancer outcomes. OC patients ≥ 50 years showed significant enrichment of *Peptoniphilus* and *Anaerococcus*, bacteria that are associated with bacterial vaginosis and chronic wound infections, where they act synergistically with other microbes to impair normal healing processes and contribute to chronic inflammatory states(56). The loss of *L. crispatus*, which maintains vaginal health through lactic acid production(16), with advancing age (3.75-fold higher in younger patients) and disease stage (5.55-fold higher in early-stage disease) suggests that protective microbiome characteristics are associated with dysbiotic shifts as both aging and cancer progress, supporting the oncobiome concept where pathogen-driven disruptions create lasting alterations promoting chronic inflammation and carcinogenesis(11). The absence of *L. crispatus* in NH-Black patients combined with higher *L. iners* prevalence represents a fundamental disparity in protective microbial capacity, as *L. crispatus* produces predominantly D-lactic acid providing superior pathogen protection compared to L-lactate from *L. iners*(58, 59). *L. iners* dominance is associated with intermediate vaginal health states and increased susceptibility to dysbiotic transitions(60), and recent mechanistic studies show that *L. iners* actively promotes treatment resistance through metabolic reprogramming(61), potentially explaining some of the documented treatment response disparities between racial groups. The combination of higher Actinomycetaceae prevalence and differential *Lactobacillus* species composition may also create a dual biological disadvantage for NH-Black patients that may contribute to worse OC prognosis. Actinomycetaceae have been associated with pelvic inflammatory disease and chronic genital inflammation(62), and recent work demonstrates that certain Actinobacteria species can metabolize estrogen and steroid hormones(63), potentially altering the local hormonal microenvironment in ways that could influence cancer biology.

Taken together, our findings highlight a potentially important role for vaginal microbiome patterns in driving OC prognosis, an area that deserves further study. Importantly, given that Black women experience later-stage diagnosis(64), more aggressive tumor biology(65), and reduced survival rates(14), differential microbiome patterns may be a potential biological mechanism that drives these disparities. Efforts to more comprehensively characterize the microbiome and identify targetable pathways may provide valuable intervention opportunities. While there is currently insufficient evidence to support routine probiotic recommendations for OC patients(66), additional studies building on our findings can provide important insights to inform species-specific rather than generic interventions to alter the microbiome.

## Limitations

Several important limitations should be considered. The cross-sectional design prevents determination of causality or temporal relationships between microbiome changes and disease characteristics. We cannot distinguish whether observed patterns reflect cancer-induced changes, treatment effects, agingrelated changes independent of cancer, or pre-existing differences influencing cancer outcomes, although the majority of patients in this cohort had received treatment.

The sample size was relatively small for detecting subtle associations, particularly for NH-Black patients (n = 22), limiting statistical power for subgroup analyses. Our 16S rRNA sequencing approach provides taxonomic composition but limited functional insight into microbiome activity. Methodological considerations such as the exclusion of patients with recent antibiotic use, and focusing on patients at least nine months post-diagnosis, potentially resulted in selecting for more stable microbiome profiles, and missing acute changes associated with initial diagnosis and treatment. The inclusion of patients across different treatment stages likely introduced heterogeneity that could have masked some associations.

Nevertheless, our study has several notable strengths that support the validity of our findings. The population-based recruitment from multiple state cancer registries enhances generalizability, while the multi-scale analytical approach combining traditional composition analysis, topic modeling, and species-level validation provided convergent evidence for our key findings. The self-collection methodology improved feasibility and reduced selection bias, and our rigorous exclusion criteria for antibiotic and suppository use minimized confounding factors.

## Conclusions

We identified consistent vaginal microbiome dysbiosis among OC patients with notable age- and raceassociated patterns that may have important implications for prognosis. These findings establish a strong foundation for future research investigating specific vaginal microbiome patterns beyond the species level in understanding and addressing OC progression and outcome disparities. If validated in larger longitudinal studies, vaginal microbiome profiles could serve as biomarkers for disease monitoring or therapeutic targets to improve outcomes, particularly for older patients and NH-Black patients who demonstrate the most concerning microbiome signatures. The integration of microbiome assessment into clinical care protocols may ultimately contribute to more personalized and effective OC management strategies, though species-specific interventions will be essential for addressing the biological disadvantages revealed by our analysis.

## Supplementary Files

This is a list of supplementary files associated with this preprint. Click to download.

- SupplementalFileMicrobiome.xlsx
- ORCHiDK01DybiosisManuscriptSupplementalNature20250821AED.pdf
- Tables.docx
